# Characteristics of Exogenous Allergen in Breast Milk and Their Impact on Oral Tolerance Induction

**DOI:** 10.3389/fped.2022.830718

**Published:** 2022-03-04

**Authors:** Chrysoula Kosmeri, Dimitrios Rallis, Maria Kostara, Vasileios Giapros, Ekaterini Siomou, Sophia Tsabouri

**Affiliations:** ^1^Department of Pediatrics, University Hospital of Ioannina, Ioannina, Greece; ^2^Neonatal Intensive Care Unit, School of Medicine, University of Ioannina, Ioannina, Greece

**Keywords:** food allergy, human milk, antigen characteristics, oral tolerance, breastfeeding

## Abstract

Food allergy is a common health problem in childhood since its prevalence was estimated to range from 6. 5 to 24.6% in European countries. Recently, a lot of research has focused on the impact of breastfeeding on oral tolerance induction. Since it was found that breast milk contains immunologically active food antigens, it would be very helpful to clarify the factors of antigen shedding that promotes oral tolerance. This narrative review aimed to summarize the latest evidence from experimental and human studies regarding allergen characteristics in human milk that may influence oral tolerance induction. A literature search in PubMed, MEDLINE, and Google Scholar was conducted. The diet of the mother was found to have a direct impact on allergen amount in the breastmilk, while antigens had different kinetics in human milk between women and depending on the antigen. The mode of antigen consumption, such as the cooking of an antigen, may also affect the allergenicity of the antigen in human milk. The dose of the antigen in human milk is in the range of nanograms per milliliter; however, it was found to have a tolerogenic effect. Furthermore, the presence of antigen-specific immunoglobulins, forming immune complexes with antigens, was found more tolerogenic compared to free allergens in experimental studies, and this is related to the immune status of the mother. While examining available data, this review highlights gaps in knowledge regarding allergen characteristics that may influence oral tolerance.

## Introduction

Food allergy is an adverse immune response to otherwise harmless food allergens that results in clinical symptoms and even in life-threatening anaphylaxis. The major food allergens are milk, egg, peanut, tree nuts, wheat, soy, fish, and crustacean shellfish (1). Food allergy has been found to affect nearly 8% of children, with evidence of increasing prevalence in recent years (2, 3). In Europe, the prevalence of self-reported food allergy ranges from 6.5 to 24.6% (4).

Through the last years, it has become evident that avoidance of food allergens in the first years of life did not lead to a decrease in food allergy prevalence, as was expected (5). On the contrary, data indicated that early introduction of food allergens had a beneficial effect in preventing food allergy in high-risk children (6), and this was more profound when allergens were consumed during breastfeeding (7). A recent meta-analysis and systematic review has concluded that maternal allergen consumption during lactation does not affect allergy risk in offspring (8). However, many available studies used maternal allergen consumption as a marker of antigen presence rather than a direct measure of antigen concentration in human milk (9).

Remarkable progress has been done regarding the evaluation of the role of breast milk in educating the developing immune system. Recent data have shown that breast milk shapes neonatal immune response through the transfer of various bioactive compounds and by influencing the composition of the gut microbiome (9–11). Since it was found that breast milk contains food antigens that are immunologically active (12–14), significant efforts have been made to establish which factors of antigen shedding in human milk can promote oral tolerance and reduction of food allergy prevalence (9, 10).

Recent reviews have proposed that the characteristics of antigens may play a role in oral tolerance induction (9, 10). In this study, a thorough literature search in PubMed, MEDLINE, and Google Scholar was done to identify the kinetics of an antigen in human milk after ingestion, the mode of allergen ingestion, and the necessary dose of an antigen to induce a tolerogenic effect. Furthermore, in the same line, the role of the specific antibodies or immunocomplexes in immune response was examined.

## Kinetics of Food Antigens in Breast Milk and Mode of Antigen Consumption

Consuming an allergen does not always lead to detectable levels of that allergen in breast milk. This is supported by the study of Metcalfe et al. (15) in which one-third of women on egg diet had no detectable ovalbumin (OVA) in their breast milk at any time through the intervention period, while in another study, OVA was detected in 28 out of 41 women on egg diet (16). This was also noticed for other allergens, Ara h1, Ara h2, and Ara h6, proteins of *Arachis hypogaea* (peanut or groundnut), which were detectable in less than half of lactating women on a peanut diet (17, 18). A possible explanation is that some women may not shed the antigen in the breast milk or that the antigen shedding may follow a different kinetic between women, so it is not always detected at the time of collection. On the other hand, women who followed an allergen avoidance diet had allergen occasionally detected in breast milk, but this might have been due to accidental ingestion of food allergens (15).

It was found that the diet of the mothers affected the antigen concentrations in breast milk if they had detectable levels of food antigens in their breast milk. This was proven for different food antigens, such as OVA (13, 15, 16), beta-lactoglobulin (BLG) (19, 20), and peanut allergens (17, 18). However, randomized controlled trials were only available for OVA. Specifically, it was proven that both the amount and the type (raw or cooked) of egg ingested had an impact on OVA concentration in human milk (15, 16). In more detail, a randomized controlled trial of 120 women found that the OVA concentration in human milk was increased 25% for each additional egg ingested per week in the first 6 weeks of lactation (15). The same result was also presented in another randomized interventional study, although it was conducted later during the 11–14th weeks of lactation. Interestingly, this study further found that cooked egg led to a higher concentration of OVA compared to raw egg (16). It was shown that cooked egg is better digested and absorbed in the small intestine than is raw egg, possibly explaining the greater concentration in human milk (21). It has also been suggested that the OVA concentration in breast milk may depend on other non-proteinic characteristics that interfere with the lipids in breast milk (22). However, the study of Palmer et al. (16) did not assess whether the allergenicity of OVA was different in women consuming raw compared to cooked eggs.

The mode of food antigen consumption may alter the allergenicity of food antigens (23, 24). In many studies, the type of cooked food antigen is not taken into consideration when assessing the tolerogenicity of the antigen. For example, in most available studies assessing peanuts as food allergens, subjects were tested using roasted peanuts (12, 17). Park et al. (25) showed that both roasted and raw peanuts had the same allergenic components; however, the processing of peanuts influences the allergenicity of peanut proteins. Specifically, the boiling of peanuts was found to decrease their allergenicity through the loss of low-molecular-weight allergens into water (26), while the roasting of peanuts was found to increase their allergenicity through the formation of neoepitopes of the main peanut allergens Ara h1 and Ara h2 (23). Therefore, it is clear that not only the mode of cooking but also food matrix interactions play a role in the allergenicity of antigens. Jeurink et al. (24) in their review, proposed that, because of these issues, mothers from different geographical locations may have different patterns of food allergens in their milk due to the different preparations of food. This is something that needs to be considered when interpreting study results.

Understanding the timing and duration of allergen shedding in breast milk is important to explain their effects on the immune response. Data regarding antigen kinetics in human milk are summarized here and are presented in Table 1. There is great variability in the kinetics of food allergens in human milk from woman to woman. The major peanut allergen Ara h6 was rapidly detected in breast milk, as soon as 10–20 min after ingestion, specifically when ingestion was on an empty stomach, indicating that consuming peanut with other food delayed the secretion of peanut proteins in human milk (12). The excretion was rapid, and the peak was early; however, the shedding was found to be long-lasting, for 24 to over 26 h (12). Most women were found to have a rapid excretion and clearance of the peanut allergens Ara h2, Ara h1, and Ara h6, while a minority of them had a more delayed excretion of Ara h2 and Ara h6 (17, 18, 27). While peanut allergens were detected at the same time in human milk, Ara h6 was in lower concentrations than Ara h2 (17). OVA was also detected in human milk within 6–8 h after ingestion (13, 16). BLG, the major allergen of cow's milk, was excreted in various rates among women, classifying them into rapid or slower metabolizers. Slower metabolizers had an allergen shedding of over a 24-h period (28). The BLG levels increased in human milk after ingestion of cow's milk and were 1.15 ng/ml on day 3 and 1.08 ng/ml on day 7 after ingestion. The mean BLG concentration in breast milk was similar in mothers of infants with cow's milk allergy and those with healthy infants. Interestingly, the peak levels of BLG were found at different times between women; Four women had peak levels in the first 24 h, 7 at 3 days, and 5 at 7 days (19). BLG may still be detected after 7 days, but this was not assessed in this study.

**Table 1:** Food allergen kinetics in human milk.

**Allergen**	**No. of women with antigen shedding/no. of participants**	**Ingestion in 1 day**	**Concentration**	**First detection after consumption**	**Peak values**	**Duration**	**Study**
Ara h6	2	30 g roasted peanuts	1–3,370 pg/ml	10–20 min	40–60 min	24 h	(12)
Ara h6	9/40	100 g roasted peanuts	1.1–79 ng/ml				(17)
Ara h6 IgG	2	30 g roasted peanuts		20 min	220 min		(12)
Ara h6 IgA	2	30 g roasted peanuts		20 min	220 min		(12)
Ara h2	9/32	100 g roasted peanuts	1–2,602 ng/ml	Rapid, 1–4 h; delayed, 8–12 h	2 h		(27)
Ara h2	14/40	100 g roasted peanuts	2.3–184 ng/ml	1–4 h (only 2 delayed excretions, 8–12 h)			(17)
Ara h1, Ara h2	11/23	50 g roasted peanuts	120–430 ng/ml	1–3 h		Rapid clearance (most <4 h)	(18)
OVA	19/41	Half a cooked egg	0.57–3.91 ng/ml	6–8 h	6–8 h		(16)
OVA	28/41	One cooked egg	1.41–4.91 ng/ml	6–8 h	6–8 h		(16)
BLG	24/47	400 ml of fat-free cow's milk	0.01–7.84 ng/ml	0–1 h	1–2 h		(28)
BLG	19	240 ml of pasteurized cow's milk	0.58–1.23 ng/ml		3 h, 24 h, 3 days, and 7 days	7 days	(19)

*BLG, beta-lactoglobulin; OVA, ovalbumin*.

The data concluded that the kinetics of food allergens was different between women and different allergens. That is to say, some allergens (peanuts) had a rapid shedding and clearance, while some others (e.g., BLG) had a more long-lasting shedding, and even some others (e.g., OVA) had a shedding of interim duration. These discrepancies could be attributed to the different study protocols. Specifically, the researchers measured the antigen concentrations in different intervals and for varied durations. Another important issue is that, although maternal diet influences antigen concentration, it is unreliable to precisely predict the presence and concentration of antigen in human milk. There is a need for more studies to assess the peak levels and the duration of shedding of different antigens in women and to assess the impact of slow or rapid shedding in oral tolerance induction.

## Dose of Antigens

Children who are breastfed are thought to be exposed to daily low doses of food allergens ingested by the mother until weaning. Food allergens are found in very small amounts in human milk in the range from picograms per milliliter to nanograms per milliliter (Table 1) (9, 15–18). Despite their low concentrations in human milk, antigens have been found able to elicit a tolerogenic response. In a study of 88 breastfed infants, OVA was detected in median concentrations of 0.15 ng/ml at 3 months and 0.173 ng/ml at 6 months of lactation. These infants had a reduced risk of egg allergy at 2.5 years of age compared to infants lactated with OVA-free breast milk (14). In the randomized controlled trial of Metcalfe et al. (15) the median OVA concentration was 0.20 ng/ml in women on a high-egg diet (>4 eggs per week) vs. 0.05 ng/ml on women on a low-egg or egg-free diet at 6 weeks of lactation. In this study, the levels of infant plasma egg-specific IgG4, a marker of possible immune tolerance, showed an average 22% increase per additional egg consumed per week. However, IgG4 was no longer detected after 16 weeks of lactation in infants receiving either a high- or a low-egg diet (15). Additionally, elimination of cow's milk from the maternal diet in the first 3 months of life was associated with lower levels of infant cow's milk-specific IgG4 and with increased risk for cow's milk allergy at 6 months of age (5).

An interesting observation is that the dose of food antigen in human milk varies widely between different individuals and different antigens (Table 1). As discussed earlier, except for the diet of the mother and the amount of antigen detected, other possible explanations for this large difference are the varying rates of antigen shedding and the different antigen kinetics between women and antigens. Moreover, Matangkasombut et al. (19) commented in their study that the variability in BLG secretion in breast milk may be due to the different rates of digestion of cow's milk, absorption, and excretion of BLG in breast milk, and they proposed that even the atopic status of the mother may affect the secretion of BLG in human milk. Available studies detected food antigens with different techniques, and this may also partly explain the differences in the results between studies. The use of sensitive and specific tests is important to detect a small number of food antigens in the lipid-rich milk matrix and to avoid cross-reactivity with other food allergens (17). Despite these hypotheses, the factors that affect the antigen concentration in human milk and the reason for the varying concentrations between women and between antigens are not fully clarified, and this is a limitation of the available studies. Future studies should address this issue and try to elucidate the factors influencing antigen concentrations between individuals.

In experimental studies, the administration of human milk containing small amounts of the peanut allergen Ara h6 and Ara h6 immune complexes in young mice led to partial oral tolerance rather than sensitization (12). This was also demonstrated in mice exposed to breast milk containing OVA in the range 180 ± 20 ng/ml, and this resulted in antigen-specific tolerance (29, 30). The amounts of OVA required for tolerance were 1,000 times lower than those needed when pups were directly fed (31, 32).

**Figure 1:**
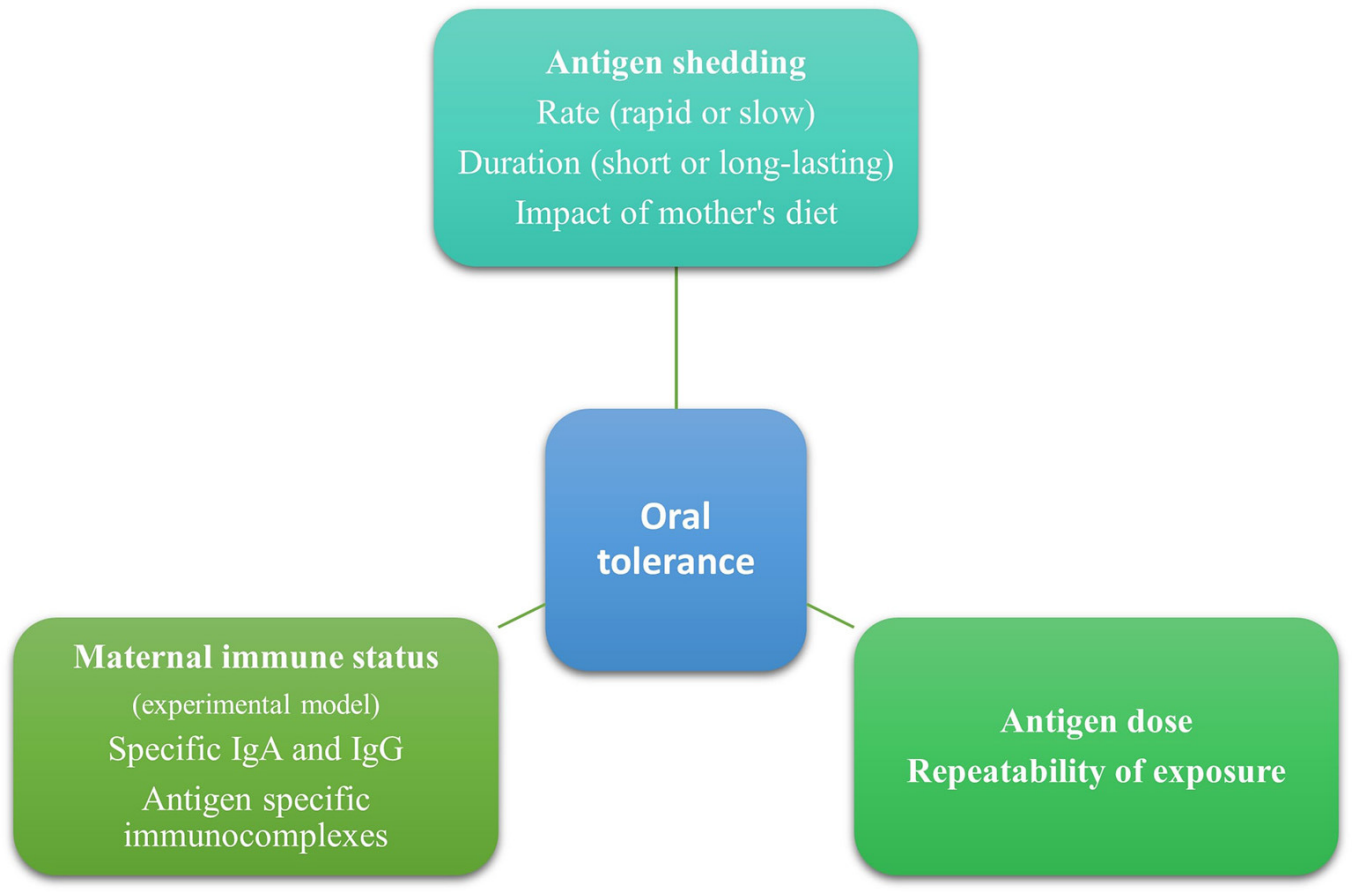
Antigen characteristics in human milk that may affect oral tolerance induction.

In animal studies, a single high dose of an antigen given orally led to sensitization, while frequent repeated low doses of oral antigens promoted tolerance (33, 34). Furthermore, immunotherapy relies on the tolerogenic effect of repeated exposures to a low dose of an antigen gradually increasing over time (35). Through breastmilk, infants are repeatedly exposed to small amounts of antigens, and this has been associated with a tolerogenic effect (Figure 1). This favors breastfeeding for acquiring oral tolerance compared to formula milk or diet, in which larger amounts of antigens are found. It would be very interesting for future studies to clarify the minimal effective dose eliciting a tolerogenic response and its duration.

## Antigen-Specific Immunoglobulins and Immune Complexes

### Animal Studies

Experimental studies showed that maternal immune status (naive, tolerant, or sensitized) to an antigen plays a role in oral tolerance induction in the offspring (36). In the study of Yamamoto et al. (37) allergic diarrhea was detected in 59.7% of mice breastfed by OVA-exposed non-sensitized mothers, in 24.6% breastfed by OVA-exposed sensitized mothers, and in 97.1% breastfed by OVA-non-sensitized and OVA-unexposed mothers. This study showed that prior sensitization of mice in conjunction with the consumption of the allergen during lactation (both sensitized and exposed mothers) provided the most potent and long-lasting protection against sensitization to this antigen in mice (38).

In experimental studies, both free allergens and specific antigen immunoglobulins were found in breast milk depending on the immune status of the mother (36, 38). Antigen-specific immunoglobulin A (IgA) and immunoglobulin G (IgG) were found in the breast milk of sensitized mothers and formed immune complexes with antigens (39, 40). In non-sensitized mothers, only free antigens were found in human milk (36). Food allergen immune complexes followed a different kinetic from that of free antigens, suggesting that they are excreted by a different mechanism (12).

Immune complexes of food antigen and maternal antigen-specific IgG in the breast milk were potent inducers of oral tolerance. OVA-specific IgG was found at significantly higher levels in milk from allergic mothers (37, 41). The large excess of OVA-specific immune complexes compared to antigen levels (100 μg/ml compared to 100 ng/ml) (37, 38) was found to be immunosuppressive (42). Antigens bound in IgA were also detected in human milk (39); however, in animal studies, IgA was not necessary for tolerance induction (29). The induction of tolerance was mediated by neonatal Fc receptor (FcRn)-dependent transfer of maternal OVA–IgG immune complexes *via* milk and the induction of allergen-specific regulatory T cells (Tregs) in offspring (38).

Similar studies were conducted for antigens other than OVA. BLG-specific IgG1 and IgA and BLG–IgG1 immune complexes were detected only in sensitized mothers, and the levels were higher with BLG exposure during lactation. Moderately sensitized mothers protected their offspring when they were exposed to cow's milk during lactation. In contrast, highly sensitized mothers provided protection against cow's milk allergy irrespective of BLG exposure (36).

Another study using human milk only from non-atopic peanut-tolerant mothers found the peanut antigen Ara h6 and Ara h6–IgG and Ara h6–IgA immune complexes, both reducing peanut-specific immune responses in lactating mice (12). However, data on peanut antigens and immune complexes are controversial. The study of Jarvinen et al. (43) proved that the transfer of maternal antibodies, the maternal immune status, and maternal exposure to peanuts had no significant impact on oral tolerance development.

### Human Studies

The levels of cow's milk antigen-specific immunoglobulins, specifically the levels of casein IgA, BLG-specific IgA, BLG-specific IgG1, and BLG-specific IgG4, were lower in human milk of mothers on a cow's milk avoidance diet compared to those with no cow's milk restriction. This could raise the speculation that avoidance of a food antigen in a mother's diet could induce food allergy. In such cases, the lower levels of cow's milk-specific IgG4 and IgA were associated with infant cow's milk allergy (5).

These observations conclude that the mother's immune status plays a role in oral tolerance induction (Figure 1). Specifically, prior sensitization of mice and the presence of immune complexes may elicit a more potent and long-lasting immune response. However, it is worth mentioning that these data originated mainly from experimental studies and should be carefully interpreted since there are differences between mice and humans. The presence and the tolerogenic effects of free antigens and immune complexes need to be further studied in humans since most mothers are tolerant of food allergens. Furthermore, human milk, other than immunoglobulins and immune complexes, contains several bioactive factors such as immune cells, antibodies, microbiota, oligosaccharides, soluble receptors, and cytokines that have a role in immune system education and oral tolerance induction (44). This should be taken into consideration when trying to elucidate factors promoting food tolerance.

## Gaps in Our Knowledge

Although a lot of research has been done on the field and there are many interesting and novel findings on breastfeeding properties and food allergy prevention, there are still some questions that need to be clarified regarding the antigen characteristics in human milk and their role in decreasing food allergy:

- What is the role of maternal diet in antigen shedding and, finally, in the concentrations of antigens in human milk?
- What is the dose range of an antigen able to promote a tolerogenic effect?
- Although the immune status of the mother affects the immune response of offspring in animal studies, is this also true in humans?
- What are the mechanisms by which the bioactive components of human milk, such as immune cells, oligosaccharides, cytokines, and soluble receptors, affect oral tolerance induction?

## Conclusion

In conclusion, the tolerogenic effect of an allergen depends on the combination of the antigen's characteristics. Antigen shedding varies between women and antigens. Notably, a small dose of an antigen can promote a tolerogenic effect. Furthermore, the presence of antigen-specific immunoglobulins and immune complexes has a positive effect on allergy prevention, indicating that the mother's immune status should be taken into consideration when trying to develop strategies for food allergy prevention. This review detects gaps in knowledge regarding antigen characteristics in human milk that need to be taken into account when considering appropriate plans for restraining the development of food allergy.

## Author Contributions

CK performed the literature search and drafted the manuscript. DR and MK contributed to literature search and drafting the work. VG substantially contributed to the design of the work and critically revised it. DR and ES critically revised the manuscript. ST proposed the writing of the article, supervised, and critically revised the work. All authors provide approval for publication of the content and agree to be accountable for the content of the work.

## Conflict of Interest

The authors declare that the research was conducted in the absence of any commercial or financial relationships that could be construed as a potential conflict of interest.

## Publisher's Note

All claims expressed in this article are solely those of the authors and do not necessarily represent those of their affiliated organizations, or those of the publisher, the editors and the reviewers. Any product that may be evaluated in this article, or claim that may be made by its manufacturer, is not guaranteed or endorsed by the publisher.
