# Corrigendum: Characteristics of Exogenous Allergen in Breast Milk and Their Impact on Oral Tolerance Induction

**DOI:** 10.3389/fped.2022.898795

**Published:** 2022-04-14

**Authors:** Chrysoula Kosmeri, Dimitrios Rallis, Maria Kostara, Vasileios Giapros, Ekaterini Siomou, Sophia Tsabouri

**Affiliations:** ^1^Department of Pediatrics, University Hospital of Ioannina, Ioannina, Greece; ^2^Neonatal Intensive Care Unit, School of Medicine, University of Ioannina, Ioannina, Greece

**Keywords:** food allergy, human milk, antigen characteristics, oral tolerance, breastfeeding

Vasileios Giapros was not included as an author in the published article. The corrected **Author Contributions** statement appears below.

## Author Contributions

CK performed the literature search and drafted the manuscript. DR and MK contributed to literature search and drafting the work. VG substantially contributed to the design of the work and critically revised it. DR and ES critically revised the manuscript. ST proposed the writing of the article, supervised, and critically revised the work. All authors provide approval for publication of the content and agree to be accountable for the content of the work.

The authors apologize for this error and state that this does not change the scientific conclusions of the article in any way. The original article has been updated.

## Publisher's Note

All claims expressed in this article are solely those of the authors and do not necessarily represent those of their affiliated organizations, or those of the publisher, the editors and the reviewers. Any product that may be evaluated in this article, or claim that may be made by its manufacturer, is not guaranteed or endorsed by the publisher.

